# Hyperinsulinemia impairs decidualization via AKT-NR4A1 signaling: new insight into polycystic ovary syndrome (PCOS)-related infertility

**DOI:** 10.1186/s13048-023-01334-8

**Published:** 2024-02-03

**Authors:** Nan-Xing Jiang, Wei-Jie Zhao, Hao-Ran Shen, Dan-feng Du, Xue-Lian Li

**Affiliations:** 1 Obstetrics and Gynecology Hospital, Fudan University, 200011 Shanghai, People’s Republic of China; 2 Shanghai Key Laboratory of Female Reproductive Endocrine Related Diseases, Obstetrics and Gynecology Hospital, Fudan University, 200011 Shanghai, People’s Republic of China

**Keywords:** Insulin, Decidualization, AKT, NR4A, Polycystic ovary syndrome

## Abstract

**Background:**

Investigating the underlying molecular mechanisms responsible for endometrial dysfunction in women with PCOS is essential, particularly focusing on the role of hyperinsulinemia.

**Methods:**

We explored the role of insulin in the decidualization process using a synthetic decidualization assay. To dissect the effects of PI3K/AKT-NR4A signaling, we employed small interfering RNAs (siRNAs) targeting the NR4A genes and inhibitors of the PI3K/AKT pathway. We also investigated the disruption of AKT-NR4A1 signaling in the endometrium of PCOS female rats induced with dehydroepiandrosterone (DHEA). Quantitative real-time PCR (qRT-PCR) and Western blot (WB) analyses were utilized to evaluate gene expression regulation.

**Results:**

Insulin was found to suppress the expression of decidualization markers in human endometrial stromal cells (hESC) in a dose-dependent manner, concurrently triggering an inappropriate activation of the PI3K/AKT pathway. Members of the NR4A family, as downstream effectors in the PI3K/AKT pathway, were implicated in the insulin-induced disruptions during the decidualization process. Moreover, the endometrium of PCOS models showed significantly elevated levels of phosphorylated (Ser473) AKT, with a corresponding reduction in Nr4a1 protein.

**Conclusions:**

Our research demonstrates that insulin negatively regulates decidualization in hESC via the PI3K/AKT-NR4A pathway. In vivo analysis revealed a significant dysregulation of the AKT-NR4A1 pathway in the endometrium of PCOS rats. These findings offer novel insights into the pathogenesis of infertility and endometrial disorders associated with hyperinsulinemia in PCOS.

## Background

Polycystic ovary syndrome (PCOS) affects 6–21% of women of reproductive age and is characterized by metabolic and reproductive anomalies, with insulin resistance (IR) and hyperinsulinemia being prominent symptoms regardless of weight [1–3]. While hyperinsulinemia is linked to the infertility seen in PCOS, it does not fully account for it, indicating the need for further exploration into other factors [4–7]. The endometrium of women with PCOS exhibits altered expression of key implantation molecules, such as LIF and HOXA-genes, which can lead to reduced receptivity and hinder decidualization [8, 9]. Inflammation markers like TNF are also elevated in obese PCOS patients, raising the risk of endometrial pathologies [10, 11].

Decidualization involves the transformation of endometrial stromal cells (ESC) into decidual cells, a process crucial for pregnancy and influenced by peptide hormones, luteal steroids, and other factors [12–14]. Defects in decidualization have been documented in PCOS, with recent research suggesting a link to hyperinsulinemia, impacting the likelihood of pregnancy after in vitro maturation (IVM) despite unaffected oocyte and embryo quality [15–17].

The Ser/Thr kinase AKT within the PI3K/AKT network is vital for various organ systems and has been implicated in decidual metabolism affected by insulin signaling [18]. The NR4A family, particularly NR4A1, NR4A2, and NR4A3, are key downstream targets in this pathway, with a potential role in the decidualization defects seen in PCOS [19–21]. Given the importance of cAMP and CREB in the process, we propose the AKT-NR4A pathway as a significant factor in insulin’s modulation of decidualization.

This article aims to elucidate how hyperinsulinemia disrupts the PI3K/AKT-NR4A pathway, leading to decidualization defects and endometrial dysfunction, thereby contributing to infertility in PCOS patients. Our preliminary findings indicate that insulin impedes decidualization in vitro, and we present first-time evidence of disrupted AKT-NR4A1 signaling in the endometrium of PCOS models [22]. These insights offer a new understanding of hyperinsulinism-related infertility in PCOS.

## Materials and methods

### Animal model

**Fig. 1:**
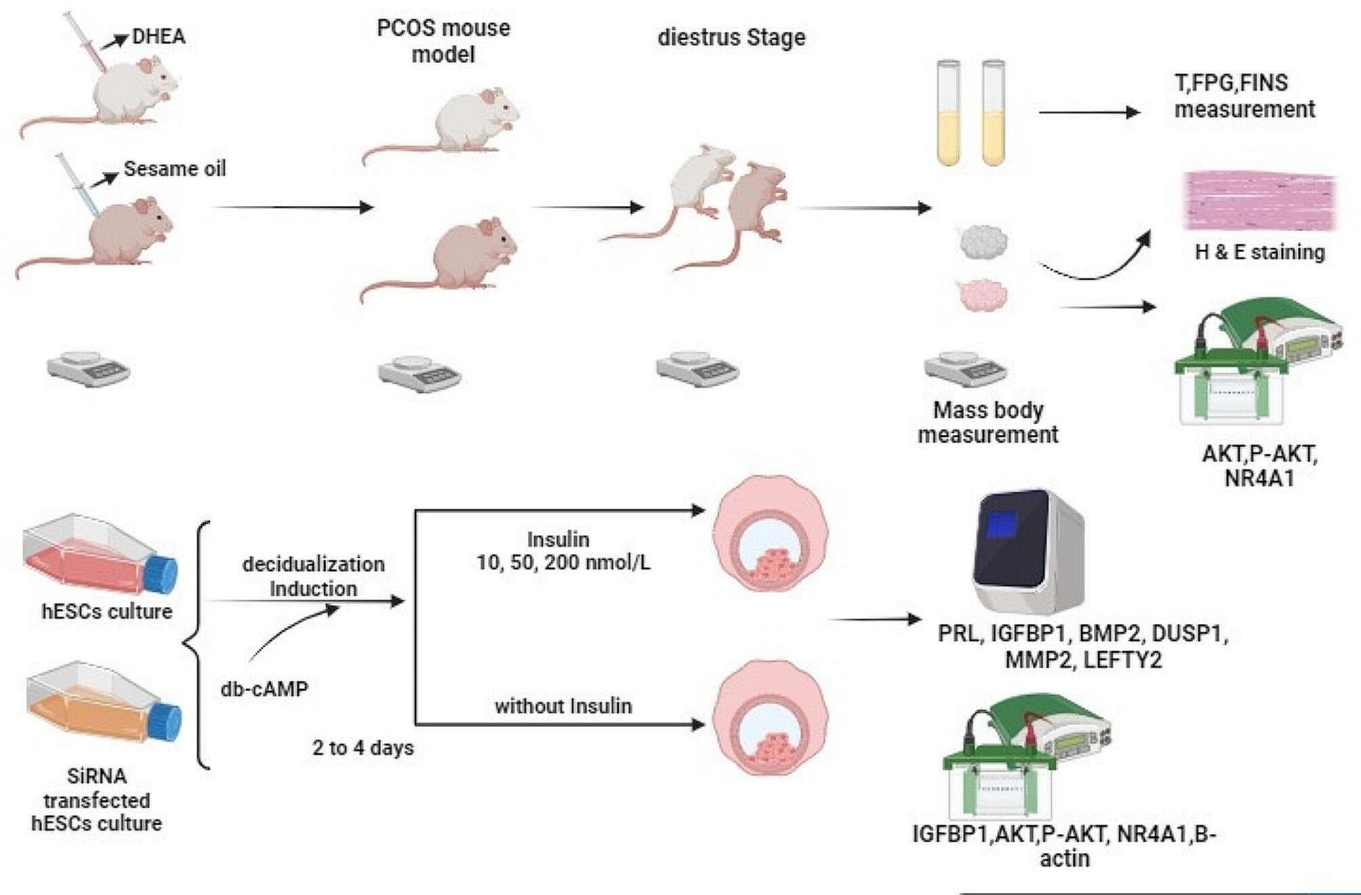
Schematic depiction of the primary steps involved in initiating a PCOS model and looking at how insulin affects human endometrial stromal cell lines (hESC)

Six-week-old female Sprague Dawley (SD) rats were maintained in a controlled environment with a 12-hour light/dark cycle and free access to food and water. The sample size was determined based on established guidelines [22]. The rats in the PCOS group (n = 8) received subcutaneous injections of dehydroepiandrosterone (DHEA) at a dose of 60 mg/kg body weight (catalogue S24516, obtained from Source Leaf Organisms, dissolved in 200µL sesame oil) daily for 21 days. In contrast, the control group (n = 5) received only sesame oil as described in a previous study [23–25]. To induce ovulation, artificial vaginocervical stimulation was performed on the rats. The phase of the estrous cycle was identified through microscopic analysis of cell types in daily vaginal smear samples [26–28]. The establishment of the PCOS model was confirmed by evaluating blood parameters and ovarian histopathology. Subsequent phases of the investigation will be conducted in accordance with the schematic representation shown in Fig. 1.

### Serum parameter assay

Blood samples were collected from the tail vein of the animals after they had fasted for 12 h, while they were still awake. Blood glucose levels during fasting were measured using an automated glucometer by Roche. Adhering to the supplied protocols, fasting insulin (FINS) levels were determined with a rodent insulin ELISA kit (Thermo Fisher Scientific, Catalog # ERINS), and testosterone concentrations were quantified using a Testosterone Parameter Assay Kit (R&D Systems, Catalog # KGE010).

### Tissue collection

In the concluding phase of the research, the body weights of all subjects were recorded. The rodents were euthanized during the diestrus phase for the collection of ovarian and uterine specimens. These tissues were then processed for various analyses, including hematoxylin and eosin (H&E) staining and protein extraction, or preserved in liquid nitrogen for future assessments.

### Ovarian morphology

Preserved ovarian tissues were sectioned at 5 micrometers thickness, discarding 40 micrometers between each section to ensure sample integrity. From each ovary, five sections were prepared, stained with hematoxylin and eosin (H&E), and then imaged at 100x magnification. Analysis was conducted using an OPTIKA microscope (M.A.D. Co. LTD, Italy). The identification of the corpus luteum and antral follicles was carried out in accordance with the criteria established in the study by Kauffman et al. [26].

### Culture conditions

The human endometrial stromal cell line (hESCs) was preserved in serum-free cell freezing medium (CELLSAVING, NCM Biotech). These cells were then cultured in DMEM/F12 medium (01-172-1ACS, Biological Industries), supplemented with 10% fetal bovine serum (FBS) (04-001-1ACS, Biological Industries) and 100 U/mL of penicillin-streptomycin (C125C5, NCM Biotech). The hESCs were maintained routinely at 37 °C in a 5% CO2 atmosphere within a humidified incubator and were subcultured at two-day intervals. For in vitro decidualization, hESCs were treated in DMEM/F12 medium with 0.5 mmol/L dibutyryl-cAMP (db-cAMP; T1418, TopScience), with or without the addition of insulin (I302196, Aladdin), for a duration of either two or four days.

### Quantitative real-time PCR (qRT-PCR)

The EZ-press RNA Purification Kit (B0004DP, EZBioscience) was employed to extract RNA from the samples. The purity and concentration of the extracted RNA were determined by measuring the OD260/OD280 ratio. Subsequently, total mRNA was converted into cDNA using the Hifair II 1st Strand cDNA Synthesis SuperMix (11120ES60, Yeasen). PCR amplifications were carried out using the Hieff UNICON Universal Blue qPCR SYBR Green Master Mix (11184ES08, Yeasen). The expression of β-ACTIN mRNA served as an internal control for normalization purposes. These procedures were replicated in three independent experiments to confirm the expression levels of the targeted genes.

### Western blot

Proteins were extracted from human endometrial stromal cells (hESC) and rat uterine tissues using ice-cold RIPA lysis buffer (WB3100, NCM Biotech) supplemented with a mix of protease and phosphatase inhibitors (HY-K0010, HY-K0021, HY-K0022, MedChem Express). Protein concentrations were quantified with a BCA Protein Assay Kit (WB6501, NCM Biotech). Equal amounts of the protein lysates were resolved on 10% SDS-PAGE gels (P2012, NCM Biotech) and subsequently transferred to polyvinylidene fluoride (PVDF) membranes (IPVH00010, Millipore). The membranes were blocked with 5% nonfat milk for one hour at room temperature, then incubated with primary antibodies targeting IGFBP1 (ab180948, Abcam), AKT (T55561, Abmart), phospho-AKT (Ser473) (Cell Signaling Technology Cat# 4060), NR4A1 (ab283264, Abcam; T56890, Abmart), and β-ACTIN (ACTB) (GB15003, Servicebio) overnight at 4 °C. This was followed by a one-hour room temperature incubation with the appropriate secondary antibodies. Band detection was performed using an ECL detection system (P10100, NCM Biotech), and band intensities were analyzed and normalized to ACTB using ImageJ software. The phosphorylation status of AKT was determined by calculating the ratio of phosphorylated AKT (Ser473) to total AKT.

### Small interfering RNA (siRNA) and transfection

Small interfering RNAs (siRNAs) from Guangzhou Ribobio, China, were utilized to suppress the expression of NR4As in human endometrial stromal cells (hESCs). The cells underwent transfection with siRNAs targeting NR4A1 (5’-GGGACTGGATTGACAGTAT-3’), NR4A2 (5’-CCACTACGCACATGATCGA-3’), and NR4A3 (5’-GTCCGTACAGATAGTCTGA-3’) using Lipofectamine 3000 (Invitrogen, Carlsbad, CA, USA). The hESCs were cultured in 6-well plates until they reached approximately 80% confluency. The medium was then replaced, and a mixture of 50 nmol/L Lipofectamine 3000 and siRNAs was gently added to each well with Opti-MEM (11,058,021, Thermo Scientific). After 24 h of incubation, the cells were assessed to confirm the efficacy of gene silencing before proceeding with further experimental procedures.

### Statistical analysis

Data are presented as the mean ± standard deviation (SD). To evaluate the differences when the data followed a normal distribution, we used the unpaired two-tailed Student’s t-test or one-way ANOVA followed by Tukey’s post hoc test. Statistical analyses were performed using GraphPad Prism version 9.3 (GraphPad Software). A P-value of less than 0.05 was considered statistically significant.

## Results

### In vitro decidualization of hESC is hindered by insulin

**Fig. 2:**
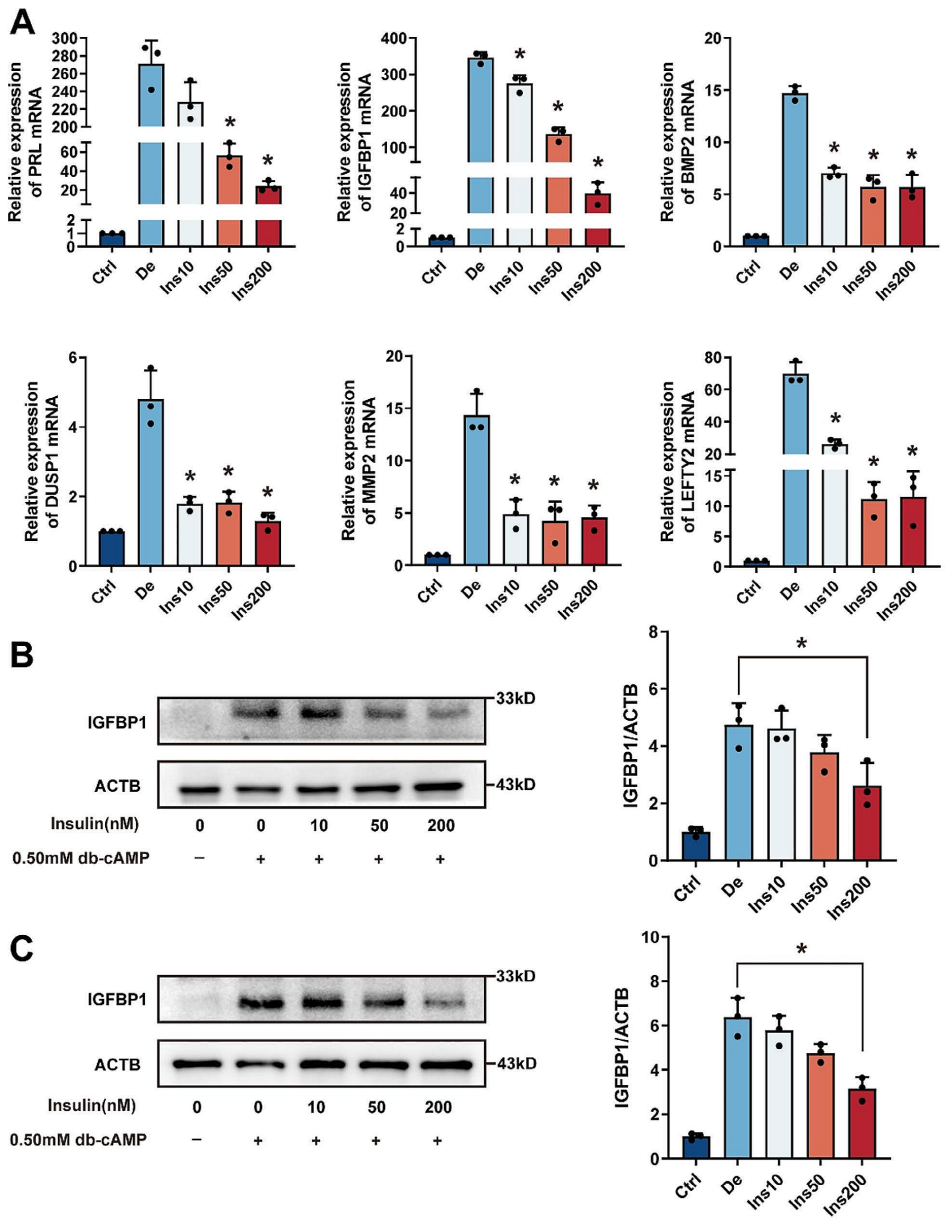
Modulation of Gene Expression by Insulin in Decidualized hESCs. (**A**) This panel displays the relative mRNA expression of PRL, IGFBP1, BMP2, DUSP1, MMP2, and LEFTY2 in human endometrial stromal cells undergoing decidualization when exposed to insulin concentrations of 10, 50, and 200 nmol/L after a 4-day period. Data are shown as mean ± SD from three independent samples. Asterisks (*) indicate a significant difference (*P* < 0.05) when compared to the differentiated control group (De). (**B**-**C**) These panels show the influence of insulin on IGFBP1 protein expression, which is induced by cAMP over 2 days (**B**) and 4 days (**C**) of decidualization. The expression levels of IGFBP1 were quantitatively assessed through densitometric analysis using ImageJ software. Results are given as mean ± SD, based on a sample size of n = 3. Significance is noted by an asterisk (*) where *P* < 0.05

Initially, to ascertain the effect of insulin on the decidualization process, human endometrial stromal cells (hESCs) were incubated with 0.5 mmol/L dibutyryl-cAMP (db-cAMP) for two or four days, with and without the presence of insulin. The successful differentiation of the cells was confirmed by the upregulated mRNA expression of specific decidual markers including prolactin (PRL), insulin-like growth factor binding protein 1 (IGFBP1), bone morphogenetic protein 2 (BMP2), dual specificity phosphatase 1 (DUSP1), matrix metalloproteinase 2 (MMP2), and left-right determination factor 2 (LEFTY2) [29, 30]. Remarkably, insulin treatment markedly reduced the mRNA expression of these decidual markers in a dose-dependent fashion in hESC (Fig. 2A). Moreover, the reduction in IGFBP1 protein levels further confirmed the inhibitory effect of insulin on decidualization when cells were treated concurrently with insulin and db-cAMP for both two and four-day periods (Fig. 2B-C). These findings suggest that elevated levels of insulin disrupt the decidualization of hESC in a laboratory setting.

### The suppressive role of insulin on hESC decidualization is conveyed through the PI3K/AKT pathway

**Fig. 3:**
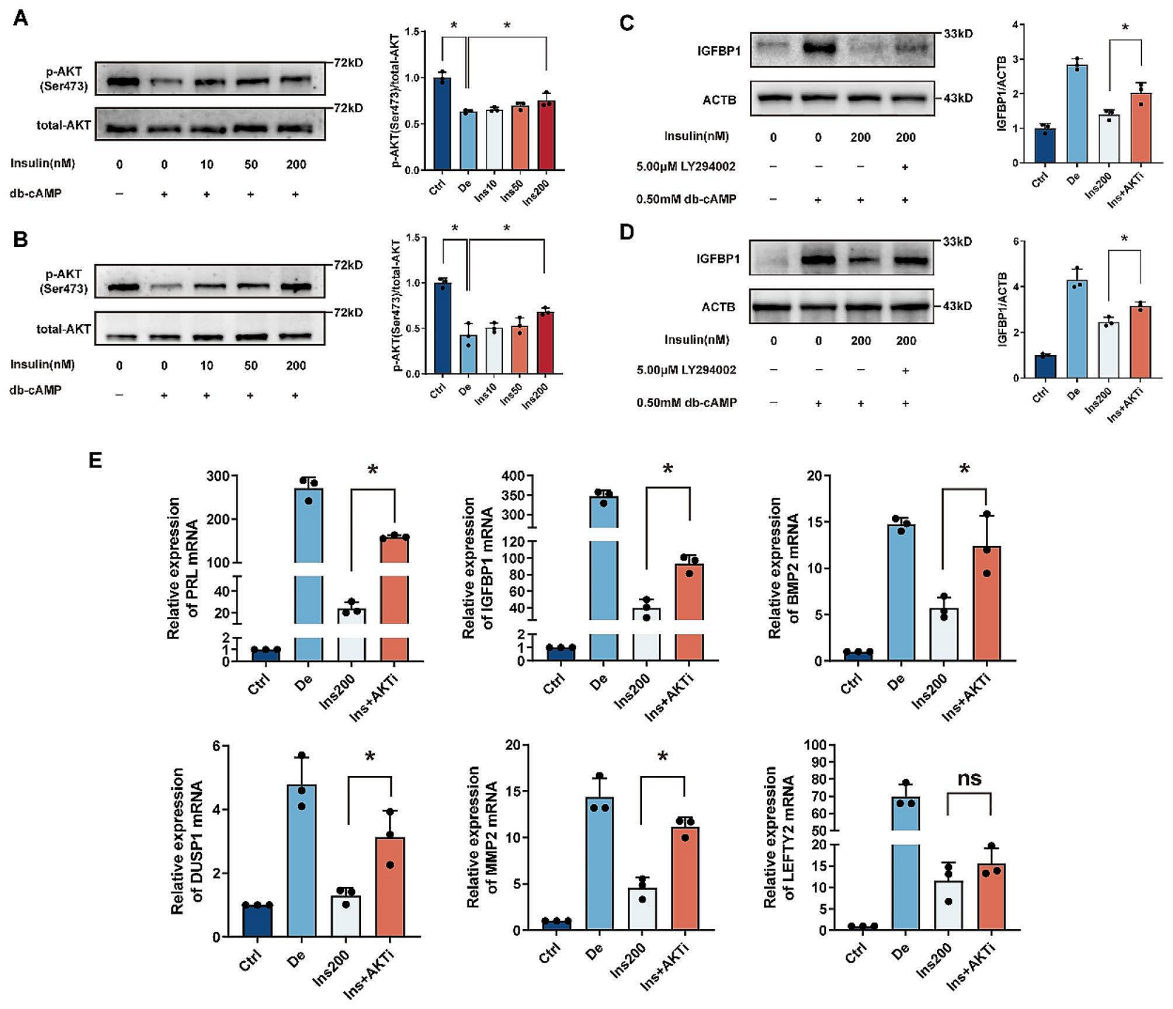
Insulin’s Suppressive Role on hESC Decidualization Operates Through the PI3K/AKT Pathway. (**A**-**B**) For a period of 2 (**A**) and 4 (**B**) days, human endometrial stromal cells (hESCs) were treated with 0.5 mmol/L db-cAMP to induce decidualization, both with and without insulin. The phosphorylation status of endometrial AKT (Ser473) was determined by Western blot analysis. (**C**-**D**) The presence or absence of the PI3K inhibitor LY294002 was factored in during the 2 (**C**) and 4 (**D**) day-decidualization period, after which the protein levels of IGFBP1 were evaluated using Western blot. Densitometric quantification of protein expression was conducted with ImageJ software. The summarized results are expressed as mean ± SD for a sample size of n = 3, with * *P* < 0.05 indicating statistical significance. (**E**) Over a 4 day interval, hESCs underwent differentiation with 0.5 mmol/L db-cAMP and 200 nmol/L insulin, with the inclusion or exclusion of 5µmol/L LY294002. mRNA expression levels were quantified by qRT-PCR. These results are also shown as mean ± SD for n = 3 samples, with * *P* < 0.05 representing statistically significant differences

While PI3K/AKT signaling is a well-recognized marker of insulin’s cellular effects [31], the specific mechanisms that link insulin’s action to the phosphorylation of AKT at Ser473 during decidualization remain poorly defined. Western blot analyses, as depicted in Fig. 3A-B, verified that phosphorylation at AKT Ser473 was diminished during the decidualization of hESC in vitro. However, insulin was observed to enhance the phosphorylation and subsequent activation of AKT Ser473 dose-dependently. The introduction of the PI3K inhibitor LY294002 mitigated the suppressive effects triggered by 200 nmol/L insulin on the decidualization of hESC. Moreover, the application of LY294002 during a two or four-day decidualization period notably increased the expression of decidual markers, with the exception of LEFTY2 (Fig. 3C-E). These results imply that insulin’s inhibitory influence on hESC decidualization in vitro is partially executed through the activation of the PI3K/AKT signaling pathway.

### The NR4A family serves as a pivotal control in hESC decidualization in vitro

The NR4A family is known to be instrumental in a host of cellular functions such as survival, programmed cell death, autophagy, immune responses, and inflammation when triggered by a variety of stimuli [32]. Particularly, NR4A1 has been identified to increase in decidualized hESC and plays a vital role in managing the expression of the decidual PRL gene in these cells, positioning it as a key transcriptional regulator in glucose metabolism [33].

**Fig. 4:**
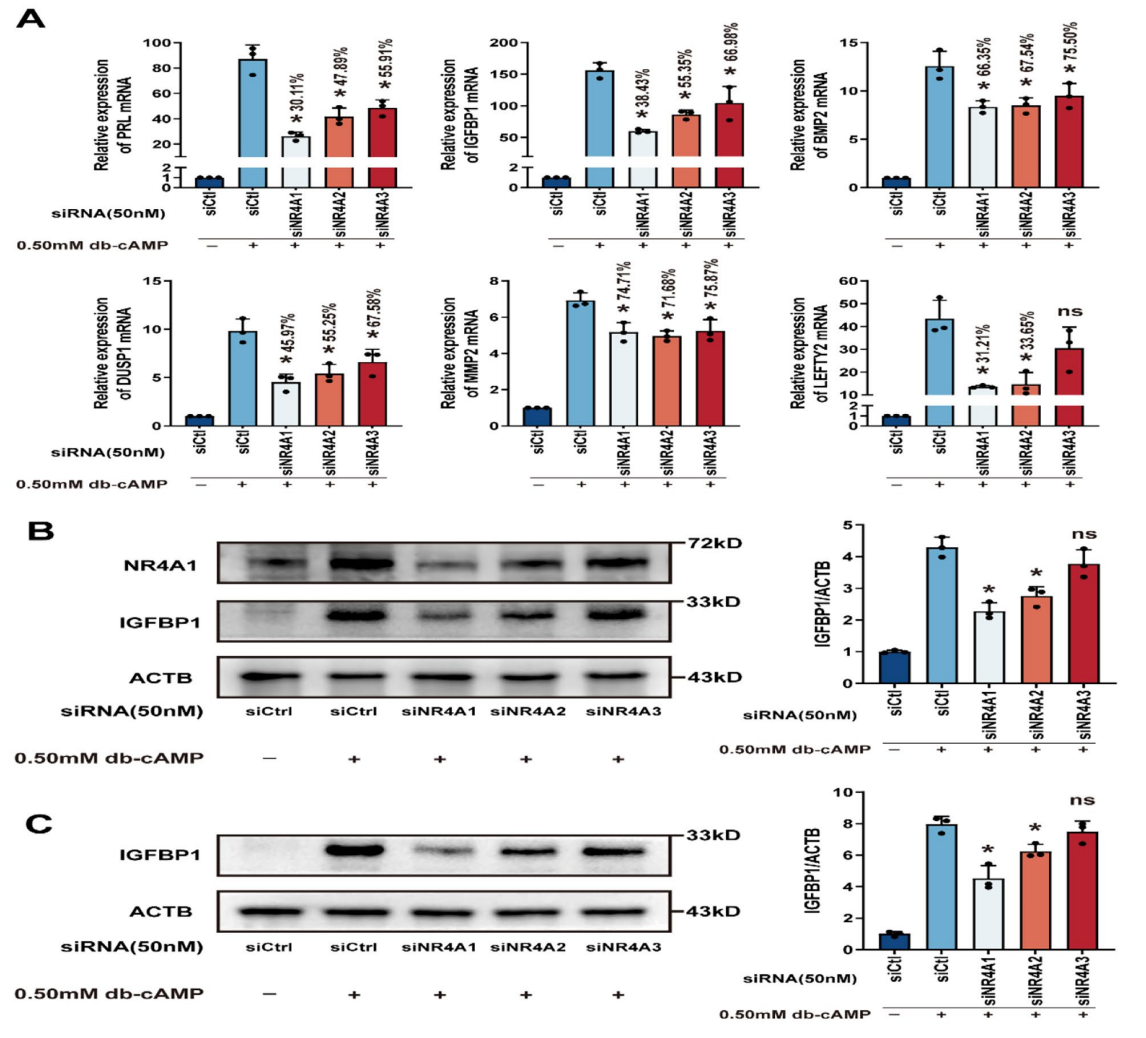
The Role of NR4As in Regulating Decidualization of hESCs In Vitro. (**A**) Human endometrial stromal cells (hESCs) underwent transfection with 50 nmol/L of specific NR4A siRNAs for 24 h, followed by an additional 4 days of treatment with or without 0.5 mmol/L db-cAMP. Subsequent qRT-PCR assays measured the relative mRNA levels of decidual markers. Presented data represent mean ± SD for n = 3 samples, with * *P* < 0.05 indicating significance relative to the siCtrl plus db-cAMP group. (**B**-**C**) The transfection’s effectiveness for the NR4A1 gene and the corresponding IGFBP1 protein levels in hESCs were treated with 0.5 mmol/L db-cAMP and targeted siRNAs for decidualization periods of 2 (**B**) and 4 (**C**) days. Protein expression was quantitatively measured using ImageJ software. The results are shown as mean ± SD for n = 3 samples, with * *P* < 0.05 denoting significance when compared to siCtrl plus db-cAMP treatment

To assess the influence of NR4As on decidualization regulation, hESCs were transiently transfected with NR4A-specific siRNAs before the initiation of the decidualization process. The subsequent qRT-PCR analyses confirmed that siRNAs targeting NR4As substantially decreased the expression of decidual markers in hESCs undergoing in vitro decidualization (Fig. 4A), suggesting the significant role of NR4A receptors in modulating decidualization, at least concerning the markers examined. The reduction of NR4A1/2 in hESCs decidualized for two or four days with 0.5mmol/L db-cAMP led to a lower expression of IGFBP1, as shown by Western blot results. In contrast, siNR4A3 had a negligible impact on IGFBP1 levels, a difference that was not statistically significant (Fig. 4B-C).

Therefore, our findings reinforce the view that the NR4A family is a crucial element in both embryo implantation and endometrial decidualization. Members of the NR4A family display significant homology in their nuclear receptor structure and genomic makeup [34]. Consequently, it is logical that all three NR4A members are engaged in the decidualization process. Nonetheless, identifying the predominant member in endometrial decidualization is challenging due to varying gene silencing efficiencies of NR4A-siRNAs in our study.

### Insulin inhibits decidualization in hESCs via AKT-NR4A

**Fig. 5:**
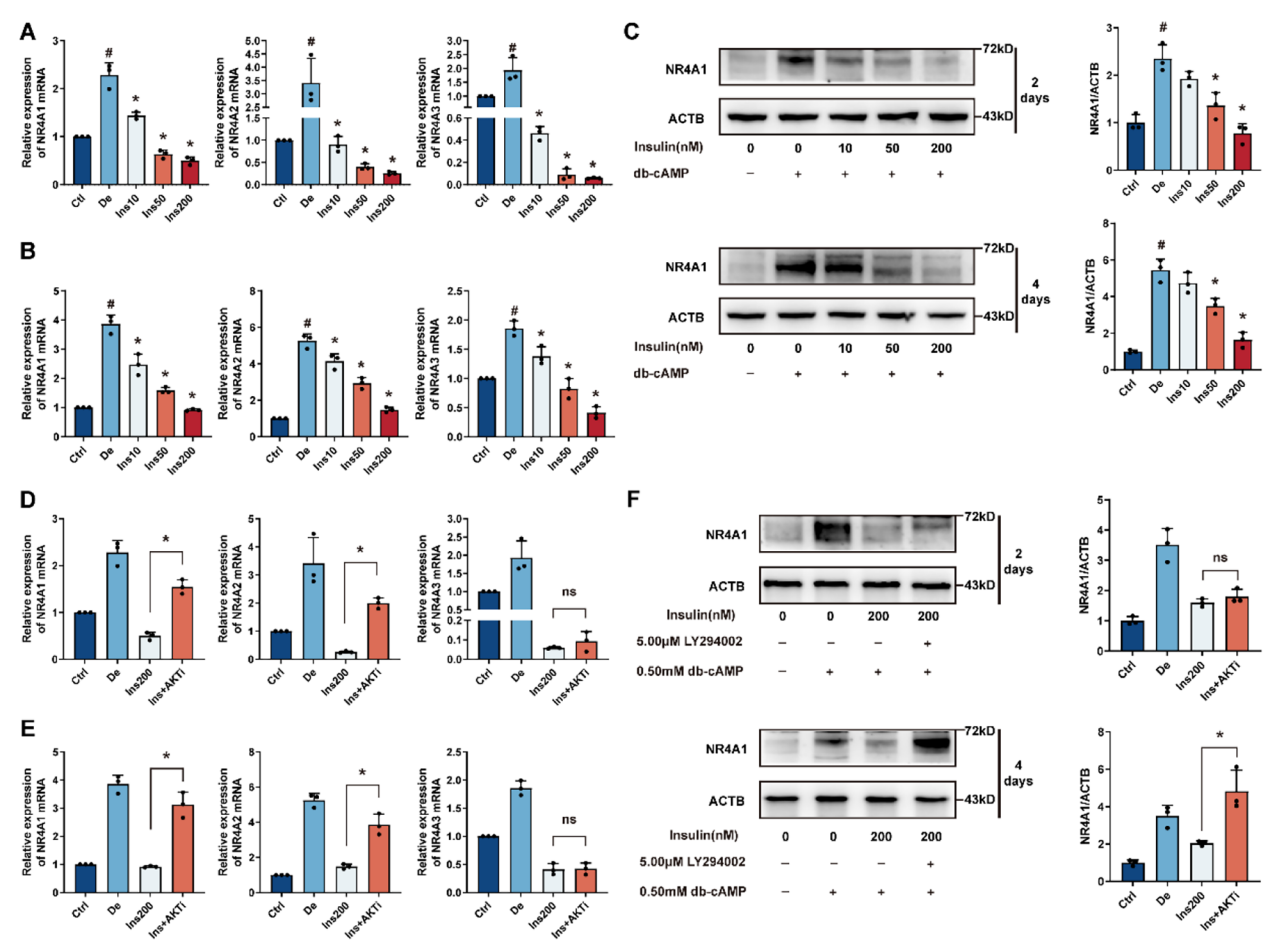
Insulin’s Inhibition of hESC Decidualization is Governed by the PI3K/AKT-NR4A Pathway. Human endometrial stromal cells (hESCs) were prompted to undergo differentiation for 2 days (**A**) and 4 days (**B**) with 0.5 mmol/L dibutyryl-cAMP (db-cAMP) and varying concentrations of insulin (0, 10, 50, 200 nmol/L). This was followed by a qRT-PCR analysis to evaluate the expression of NR4As. (**C**) Corresponds to the cell treatment detailed in (**A**), after which the cells were lysed for Western blot analysis. These results are displayed as mean ± SD for n = 3 samples, where # *P* < 0.05 indicates statistical significance compared to the undifferentiated control (Ctrl), and * *P* < 0.05 is in relation to the differentiated control (De). In a continuation of the experiment, hESCs were induced to differentiate for 2 (**D**) and 4 (**E**) days with 0.5mmol/L db-cAMP alongside 200nmol/L insulin, with and without the addition of 5µmol/L LY294002. mRNA expression was quantified by qRT-PCR and NR4A1 protein levels were determined via Western blot (**F**). These findings are expressed as mean ± SD for n = 3 samples, with * *P* < 0.05 denoting statistical significance

As delineated by Pekarsky et al. (2001), NR4A1, functioning as an early response gene, plays a pivotal role in conveying anti-apoptotic and pro-survival messages within the PI3K-AKT pathway [21]. Our investigation into the possible link between insulin and AKT-NR4As during decidualization involved measuring NR4As expression in conditions with and without insulin. Treatment with db-cAMP significantly enhanced the mRNA levels of NR4As in hESC. In contrast, insulin was observed to inhibit the db-cAMP-mediated increase in NR4As mRNA expression in a dose-dependent manner (Fig. 5A-B). Given that NR4A1 is among the initial genes to be upregulated during cAMP-stimulated decidualization in hESC [35], Western blot analysis was used to further assess the NR4A1 protein as a representative of the NR4A family. Decidualization led to raised levels of NR4A1 protein, but this increase was notably reduced at higher insulin concentrations (50 nmol/L and 200 nmol/L) (Fig. 5C). Subsequent treatment of hESCs with 5µmol/L of the PI3K/AKT inhibitor LY294002 revealed a marked elevation in the mRNA expression of NR4A1 and NR4A2 during decidualization in vitro. However, NR4A3 did not exhibit a similar upregulation when compared to cells treated with 0.5mmol/L db-cAMP and 200nmol/L insulin (Ins 200), suggesting that NR4A1 and NR4A2 are more integral to PI3K/AKT-NR4A signaling than NR4A3. Additionally, LY294002 had an effect on NR4A1 protein expression, although not significantly so for the 2-day decidualization period (Fig. 5F). In summary, the findings presented in this section suggest that the PI3K/AKT-NR4A pathway plays a role in the adverse impacts of insulin on the decidualization process in hESC.

### Activation of akt in endometrium of PCOS rats

**Fig. 6:**
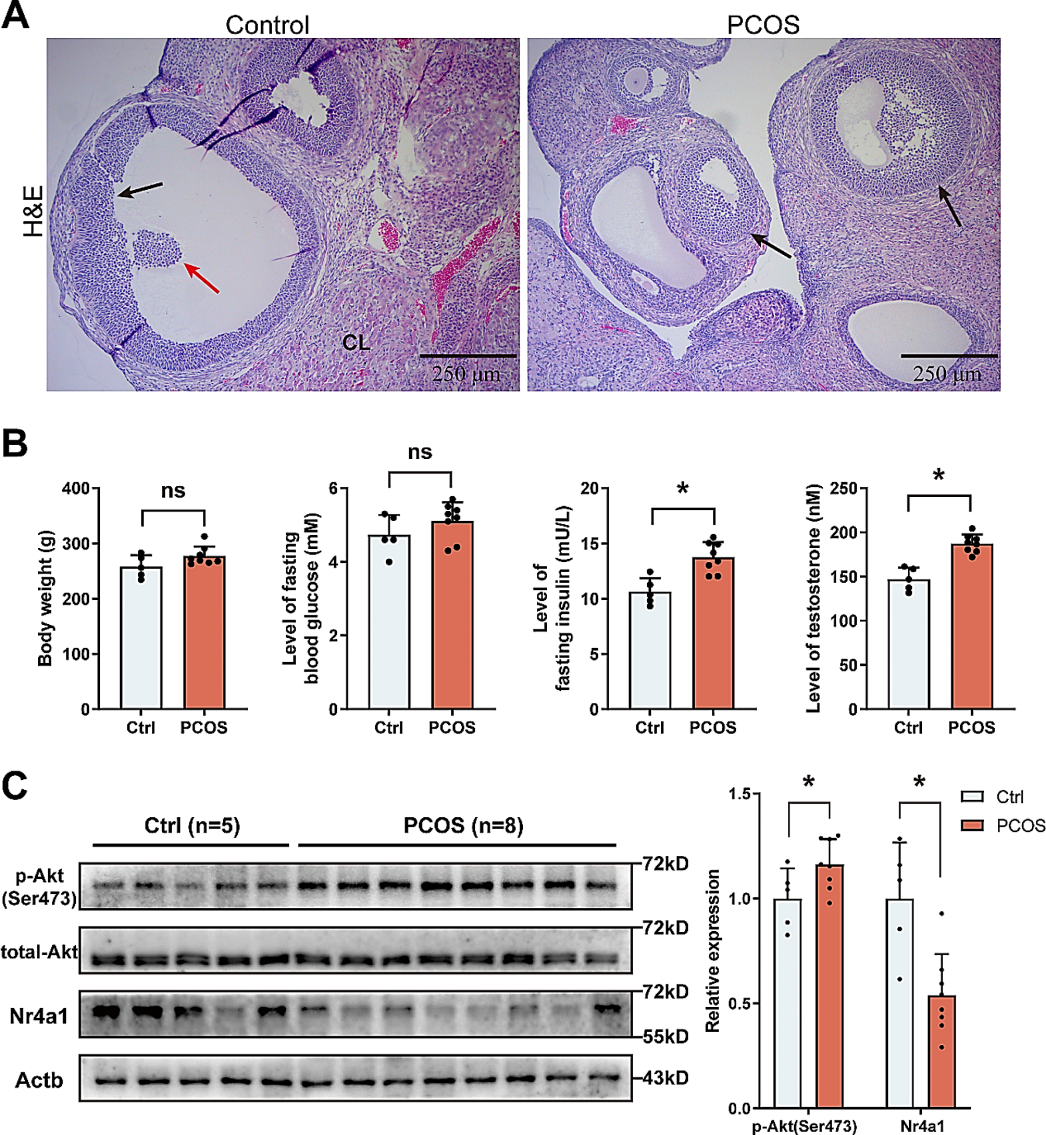
Details of the disruption of Akt-Nr4a1 signaling in the endometrium of rats with PCOS. (**A**) Depicts the alterations in ovarian morphology between the control group and the PCOS group. The corpora lutea (CL) are identified; black arrows point to the layers of granulosa cells; red arrows highlight the oocyte. (**B**) Illustrates the comparative body weight and serum parameters between the groups, with statistical significance indicated by * *P* < 0.05. (**C**) Showcases the Western blot analysis performed on total protein extracts from endometrial samples (n = 13). Quantitative densitometric evaluations of protein expression was conducted using the ImageJ software, with significant differences marked by * *P* < 0.05

The findings elucidated a fundamental molecular mechanism linked to hyperinsulinemia. Subsequently, we explored whether this mechanism could extend to the context of PCOS. Ovarian analysis of PCOS-afflicted female rats displayed polycystic features with a higher count of antral follicles compared to the control group. Notably, the absence of corpora lutea (CL) in PCOS rat ovaries pointed to ovulatory disruption (Fig. 6A). Body weight and fasting blood glucose (FBG) levels did not significantly differ between the groups (Fig. 6B). However, the PCOS group exhibited significantly increased levels of testosterone and fasting insulin (FIN), aligning with the established metabolic disturbances characteristic of PCOS. Western blotting revealed elevated phosphorylation of endometrial Akt (Ser473) in PCOS rats during the diestrus phase, along with a corresponding reduction in Nr4a1 protein (Fig. 6C). This pattern indicates a malfunction in the Akt-Nr4a1 signaling within the endometrium of PCOS rats.

## Discussion

**Fig. 7:**
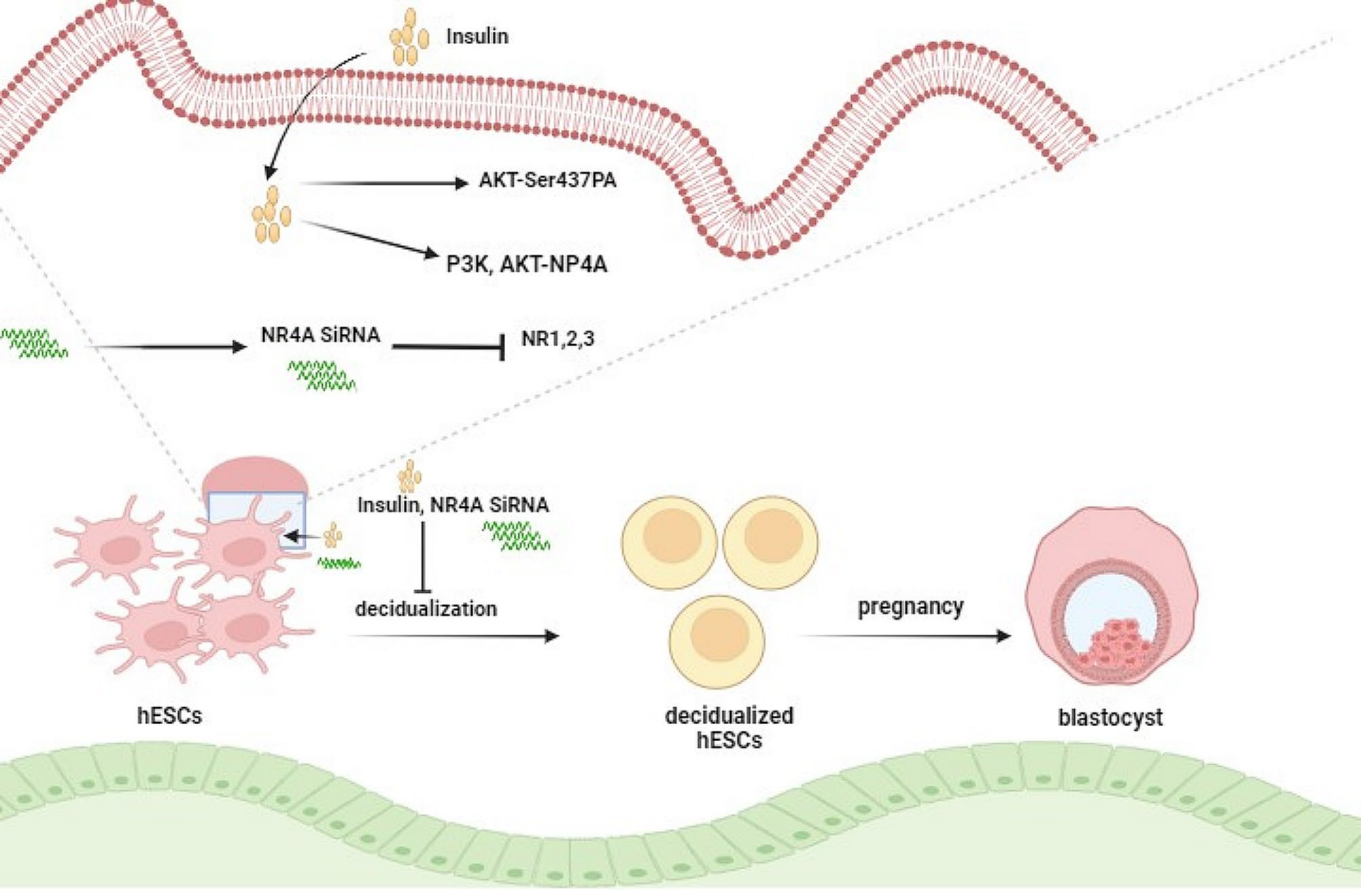
Conceptual Illustration of Hyperinsulinemia’s Impact on Decidualization Through PI3K/AKT-NR4A. The diagram depicts how insulin activates PI3K/AKT signaling, which in turn suppresses the expression of NR4A1/2, culminating in hindered decidualization

The successful union of the embryo, endometrium, and their interaction is a cornerstone for reproductive success in humans [36]. While it is widely held that infertility associated with PCOS is primarily due to irregular or absent ovulation [37, 38], and while initiating ovulation remains a key treatment strategy [39], research by Palomba et al. suggests that infertility risk persists even for ovulating PCOS individuals or those with medically induced ovulation, highlighting a possible “endometrial factor” in play [40]. In PCOS, the endometrium presents distinctly altered expressions in genes related to energy metabolism, angiogenesis, and inflammation, affecting its receptivity [41]. Hyperinsulinemia is a major component contributing to PCOS’s complexity and is intimately linked to infertility and recurrent pregnancy loss [42, 43]. Yet, systematic studies into endometrial insulin signaling and its downstream effects have been scarce. Our study uncovers how insulin disrupts decidualization via the PI3K/AKT-NR4A pathway in hESC for the first time. We also observed significant dysregulation in AKT (Ser473)-NR4A1 signaling in the endometrium of PCOS rats (Fig. 7).

Insulin’s modulation is vital for optimal endometrial functions, including proliferation, decidualization, and embryo implantation [44, 45]. The growing understanding of hyperinsulinemia’s impact on endometrial functions underscores the need to deepen our knowledge of its role in the reproductive capabilities of women with PCOS [42]. Insulin impacts the endometrium through its receptor and, at higher concentrations, through the IGF-IR [46]. AKT signaling, critical to insulin’s cellular responses, requires dual phosphorylation at Thr308 and Ser473 for full activation [46]. Our findings corroborate that decidualization in hESC is correlated with the deactivation of Ser473-AKT, in line with prior studies [47]. Interestingly, our results further show that hyperinsulinemia significantly suppresses decidual markers, an effect that can be partially reversed by inhibiting Ser473-AKT phosphorylation.

The PI3K/AKT pathway influences numerous downstream substrates, including the FOXO family, which plays a central role in endometrial homeostasis during the menstrual cycle and early pregnancy [48–50]. In decidualizing hESC, insulin mediates FOXO1’s transcriptional activity via PI3K [51]. Previous studies have also shown that AKT can phosphorylate NR4A1, leading to its inactivation as a transcription factor [21, 34]. This suggests that NR4As are adaptable downstream targets of AKT, shaping various cellular functions. NR4A receptors, crucial in regulating glucose and lipid homeostasis among other processes, are subject to post-translational modifications [20, 34, 52]. When activated by growth factors, they undergo phosphorylation at serine residues [21, 34]. Notably, NR4A1 has been spotlighted for its role in endometrial function, essential for progesterone-mediated endometrial changes prior to embryo implantation [29] and associated with decidualization in hESC [33]. Our study confirms that NR4A subfamily expression is induced by decidualization stimuli in hESC, and silencing these receptors markedly downregulates decidual markers [53]. However, the exact function of NR4As in decidualization remains to be further clarified.

Our subsequent in vitro experiments in hESC demonstrated that insulin-induced decidualization impairment and downregulation of NR4A1/2 are mediated by PI3K/AKT activation, a process that can be alleviated with the PI3K inhibitor LY294002. Thus, NR4A1 and NR4A2 act as metabolic transmitters of insulin signals in the PI3K/AKT pathway during hESC decidualization. Furthermore, phosphorylated endometrial Akt (Ser473) activation was observed in PCOS rats, with concurrent Nr4a1 protein reduction, highlighting the pivotal role of Akt (Ser473)-Nr4a1 in the insulin signaling pathway during decidualization.

Metabolic disturbances, particularly IR and hyperinsulinemia, are prevalent in most PCOS women [54]. Hyperinsulinemia can both result from and contribute to IR [55], and it prominently occurs in skeletal muscle, liver, and adipocytes [31, 56]. In those with insulin resistance, more insulin is required to manage elevated blood glucose levels, which also leads to impaired insulin action in other tissues, including the endometrium. Moreover, metabolic and inflammatory alterations in the endometrium of PCOS patients may further exacerbate endometrial dysfunction. The dysfunction may include reduced endometrial receptivity and decidualization, which could be a factor in the subfertility experienced by pregnant PCOS women [57]. Differentiating the effects of insulin from those of androgens in the endometrium is complex due to their interrelated increase in PCOS [58]. A hyperandrogenic environment in the human endometrium alters gene expression related to insulin signaling, glucose metabolism, and transport [59]. Furthermore, heightened inflammatory markers such as TNF inhibit IRS1 activation and glucose transport in the endometrial stromal cells of PCOS patients, with testosterone and insulin intensifying these effects [10].

A nuanced understanding of these metabolic abnormalities is essential for addressing the underlying endometrial dysfunction in PCOS. Our findings suggest a critical relationship between the dysregulation of insulin signaling pathways and endometrial function, which could have profound implications for the treatment and management of PCOS-related infertility. The intricate balance of metabolic and signaling pathways in the endometrium highlights the need for targeted therapeutic strategies that address both the systemic and local effects of PCOS. Also, women with PCOS have been found to have alterations in their gut microbiome composition, known as dysbiosis. Therefore, dysbiosis modulating treatments can be useful for these patients as well [60, 61].

Our study has yielded pivotal insights into the complex relationship between insulin signaling and endometrial function, particularly in the context of infertility associated with Polycystic PCOS. These insights compel us to reconsider clinical approaches and tailor them more precisely to the individual’s needs. The revelation that the PI3K/AKT-NR4A signaling pathway is instrumental in the function of both hES and the endometrium of PCOS-affected rats underscores the potential for personalized medical strategies. By targeting this pathway, we can directly address the specific molecular anomalies that undermine proper decidualization in PCOS, paving the way for an approach to infertility treatment that is fine-tuned to the patient’s unique molecular profile. Historically, the management of PCOS-related infertility has largely centered around stimulating ovulation. However, our findings illuminate the pressing need to expand this focus and recognize the substantial role of the endometrium in successful reproduction. It becomes clear that assessments of endometrial function should be integrated into the diagnostic and treatment protocols for PCOS, moving us past an exclusive focus on ovulation and towards a more holistic understanding of fertility. By deciphering the disruption of insulin signaling through the PI3K/AKT-NR4A pathway, we uncover a promising avenue for therapeutic intervention. Tailoring treatments to rectify this specific dysfunction offers hope for improving endometrial receptivity, addressing a key barrier to fertility that many women with PCOS face. Furthermore, the intricate role of insulin in endometrial function invites us to think more broadly about long-term reproductive health in PCOS. Clinicians should develop strategies aimed at maintaining insulin sensitivity and managing hyperinsulinemia throughout a woman’s reproductive years to support overall fertility health. This complex scenario mandates a coordinated, interdisciplinary approach. Endocrinologists, gynecologists, fertility specialists, and reproductive biologists must collaborate closely to navigate the intricate hormonal, metabolic, and endometrial interplays that characterize PCOS-related infertility. Integrating these insights into clinical practice also calls for robust patient education. Equipping individuals with PCOS with a clear understanding of how insulin impacts their endometrial function is crucial. Knowledge empowers them to be active participants in their care, making informed decisions alongside their healthcare providers in a supportive, cooperative environment. In essence, our research advocates for a fundamental shift in the clinical management of PCOS-related infertility. By embracing the complexity of the molecular underpinnings revealed in our study, healthcare professionals can transcend the traditional boundaries of ovulation induction. Such a paradigm shift encourages the adoption of targeted and personalized management strategies, enhancing fertility outcomes for women with PCOS.

## Conclusion

In summary, our research has for the first time demonstrated that insulin disrupts the process of decidualization in human endometrial stromal cells (hESC) through the PI3K/AKT-NR4A pathway (Fig. 7). Moreover, we have shown that there is a significant dysregulation of the Akt-Nr4a1 pathway within the endometrium of PCOS rats, offering fresh insights into the underlying mechanisms of infertility linked to hyperinsulinism in PCOS. It is important to highlight that our focus was primarily on the NR4A1 protein, as the NR4A family exhibits conserved structural domains within their genomic sequence. NR4As are known for their swift response as early-response genes and their transcriptional activity is regulated by posttranslational modifications. For instance, phosphorylation at Ser350 and Ser354 in the DNA-binding domain of NR4A1 inhibits its transactivation capability [34]. However, this study was not designed to discern changes in phosphorylated NR4As. Although preliminary, our findings contribute a novel perspective to the understanding of infertility and endometrial dysfunction in women with PCOS who are experiencing hyperinsulinemia.

## Data Availability

The original contributions presented in the study are included in the article.
